# Strawberry Vein Banding Virus P6 Protein Is a Translation Trans-Activator and Its Activity Can be Suppressed by FveIF3g

**DOI:** 10.3390/v10120717

**Published:** 2018-12-15

**Authors:** Shuai Li, Yahui Hu, Lei Jiang, Penghuan Rui, Qingqing Zhao, Jiying Feng, Dengpan Zuo, Xueping Zhou, Tong Jiang

**Affiliations:** 1 School of Plant Protection, Anhui Agricultural University, Hefei 230036, China; 18356086590@163.com (S.L.); yahui1990@outlook.com (Y.H.); jianglei062x@ahau.edu.cn (L.J.); 15755308130@163.com (P.R.); zqqsvbv@163.com (Q.Z.); raapidrapidrapid@sina.com (J.F.); vvblackstarxixi@163.com (D.Z.); 2 State Key Laboratory for Plant Disease and Insect Pest, Institute of Plant protection, China Academy of Agricultural Sciences, Beijing 100193, China; zzhou@zju.edu.cn

**Keywords:** strawberry vein banding virus, P6 protein, trans-activate, bicistron, FveIF3g

## Abstract

The strawberry vein banding virus (SVBV) open reading frame (ORF) VI encodes a P6 protein known as the RNA silencing suppressor. This protein is known to form inclusion like granules of various sizes and accumulate in both the nuclei and the cytoplasm of SVBV-infected plant cells. In this study, we have determined that the P6 protein is the only trans-activator (TAV) encoded by SVBV, and can efficiently trans-activate the translation of downstream *gfp* mRNA in a bicistron derived from the SVBV. Furthermore, the P6 protein can trans-activate the expression of different bicistrons expressed by different caulimovirus promoters. The P6 protein encoded by SVBV from an infectious clone can also trans-activate the expression of bicistron. Through protein-protein interaction assays, we determined that the P6 protein could interact with the cell translation initiation factor FveIF3g of *Fragaria vesca* and co-localize with it in the nuclei of *Nicotiana benthamiana* cells. This interaction reduced the formation of P6 granules in cells and its trans-activation activity on translation.

## 1. Introduction

Strawberry vein banding virus (SVBV) is an important plant virus infecting strawberries. SVBV is now widely distributed in many countries worldwide, including Australia, Japan, multiple European countries, The United States of America, and China, and causes severe loss of strawberry production [1,2,3]. SVBV is known to be transmitted by several aphid species, including *Chaetosiphon* sp., in a semi-persistent manner [4]. Symptoms of SVBV infection in strawberry (*Fragaria×ananassa* cv. Sachinoka) plants are mainly retarded plant growth and fewer numbers of creeping stems [5]. The woodland strawberry (*Fragaria vesca*) is a diploid wild strawberry species that has been used as an indicator plant of SVBV infection assays. Symptoms of SVBV-infected *F. vesca* plants are mainly chlorosis along the leaf veins and lobular distortion [6]. When *F. vesca* plants are mix-infected with SVBV and Strawberry crinkle virus (SCV), Strawberry mottle virus (SMoV), Strawberry mild yellow edge virus (SMYEV), or Strawberry latent ring spot virus (SLRSV), the infected plants often show leaf distortion, chlorosis, plant stunting, and fruit yield reduction [7,8].

**Figure 1:**
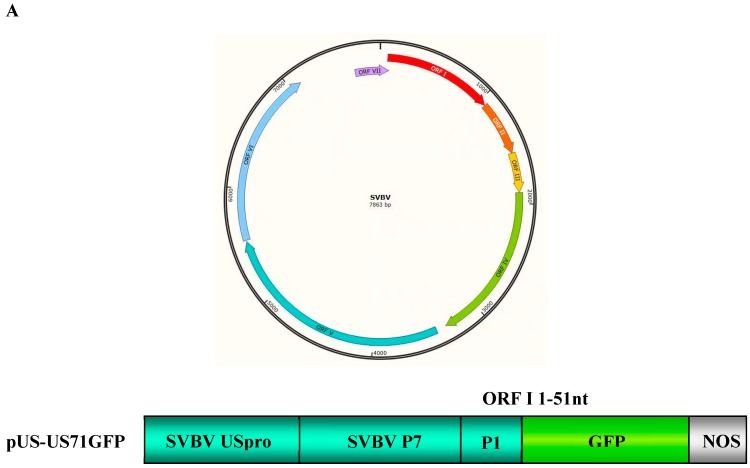

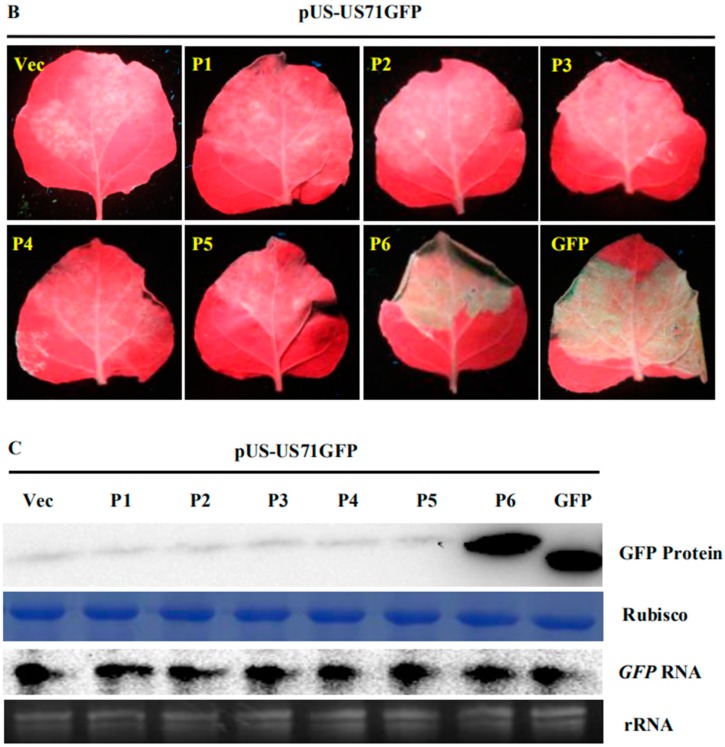
Strawberry vein banding virus (SVBV) P6 is the only translation trans-activator; (**A**) map of the SVBV genome and bicistron construct. The ring symbolizes the 7863-bp double-stranded SVBV DNA and the arrangement of seven open reading frames (ORFs). US-US71GFP contains the SVBV-US isolate promoter (pUS) and a fused p7-p1-gfp fragment; (**B**) leaves were co-infiltrated with pUS-US71GFP and the empty vector (Vec, pCAM2300), pCAM-USP1 (P1), pCAM-USP2 (P2), pCAM-USP3 (P3), pCAM-USP4 (P4), pCAM-USP5 (P5), pCAM-USP6 (P6), or pCAM-GFP (GFP). The infiltrated leaves were photographed under the UV illumination at 3 days post agro-infiltration (dpai); (**C**) Western blot and Northern blot assays of the GFP protein and GFP mRNA accumulation in various infiltrated leaves. P1 through P6 indicate the six vectors expression SVBV P1, P2, P3, P4, P5 and P6 proteins. The same samples were used for these two assays. Western blot was probed with a GFP specific monoclonal antibody and the Northern blot was probed with a DIG-labeled GFP-specific probe. Coomassie blue staining was used to estimate protein loadings and ethidium bromide staining was used to estimate RNA loadings.

SVBV is a member of the genus *Caulimovirus*, family *Caulimoviridae*. The SVBV genome is a double-stranded DNA (dsDNA) with about 8000 nucleotides [9]. SVBV genomic DNA has seven open reading frames (ORFs) and shares similar genome structure with Cauliflower mosaic virus (CaMV) (Figure 1A) [5]. The CaMV ORF VI-encoded P6 protein was reported as a multifunctional protein [10]. For example, CaMV P6 can function as an RNA silencing suppressor, and is the main component of CaMV inclusion bodies [11,12]. It is also known to be a disease symptom and host range determinant [13]. In addition, P6 was reported to act as a trans-activator, to interact with eukaryotic cell translation initiation factors eIF3g, L18, and L24 proteins of the 60S ribosomal subunit, and to specifically control the synthesis of downstream proteins from CaMV 35S RNA [14]. Finally, the CaMV P6 protein was also shown to regulate the expression of multiple genes in its host plants [15]. In our previous studies, the SVBV P6 protein had also been identified as an RNA silencing suppressor, which could effectively enhance the expression of the exogenous GFP gene *Nicotiana benthamiana* [11].

Translation in eukaryotic cells begins with the 43S ribosomal complex, which includes a 40S ribosome small subunit, eIF1, eIF1A, eIF3, and eIF2/GTP/Met/tRNA complex. The 43S ribosomal complex starts to scan and recognize the 5′-untranslated region of mRNA. After recognizing the initiating codon of the first ORF, the 43S and 60S ribosomal subunits assemble to form 80S ribosomes and to initiate RNA translation [16]. The translation initiation process for plant virus proteins is relatively simple. In order to achieve efficient virus replication and virion assembly in host cells, the virus generally needs to regulate the transcription and translation of its various ORFs by two main strategies. One strategy is cis-regulation, which needs cis-acting elements such as promoters and enhancers that are directly important for the expressions of their own and other functional genomes [17]. The other strategy is trans-regulation, which regulates the expressions of multiple virus proteins through its transcription or translation products such as the trans-activate protein (TAV). The expression of host related proteins can also be regulated to be convenient for virus replication and assembly [18]. The ribosome initiation mechanism is also used by viruses to control viral RNA translation. After sliding through the termination codon of the previous ORF, the ribosome continues to translate the next gene by forming a complex with the viral TAV and other necessary translation co-factors [19].

The ribosome translation initiation mechanism has been reported for multiple plant viruses in the family *Caulimoviridae*, these being CaMV, Figwort mosaic virus (FMV), and Peanut leaf streak virus (PCSV) [20,21]. CaMV35S RNA translation can be re-initiated via two different mechanisms. First, the ribosome re-initiates ORF VII translation through a diversion mechanism until the synthesis of 35S RNA leader sRNA is completed, and the translation efficiency can be enhanced by 2–3 fold in the presence of a TAV [22]. Second, CaMV transcribes 19S RNA and 35S RNA from its genomic DNA using RNA polymerase II. The 19S RNA contains only the ORF VI region and produces a TAV protein (also known as the P6 protein) [23]. SVBV 35S RNA is a polycistron RNA and encodes five proteins (i.e., P1, P2, P3, P4, and P5) from the ORF I, II, III, IV, and V. The P6 protein is a highly expressed protein and accumulates in the cytoplasm, forming cellular inclusion-like granules [24]. CaMV P6 can also interact with the 60S polyribosome subunit proteins L18, L24, eukaryotic initiation factor eIF3g, and re-initiation scaffolding proteins (RISP) to form complexes [25] to translate viral 35S polycistron RNA continuously [26].

In this study, we show that the SVBV-encoded P6 protein is the only TAV of the virus and can trans-activate the translation of downstream cistrons, as well as the expression of GFP from various vectors carrying different caulimovirus promoters and bicistrons. In addition, the P6 protein encoded by SVBV expressed from an infectious clone could also trans-activate the translation of bicistrons. The FveIF3g protein of *F. vesca* could interact with the P6 protein in nuclei, interfere with the formation of P6 granules in plant cells, and inhibit the trans-activation activity of P6. Our findings revealed a potential molecular mechanism controlling the translation of viral polycistron RNA assisted by the P6 protein. Furthermore, the findings provided a direction for future research on double stranded DNA (dsDNA) virus replication and translation.

## 2. Materials and Methods

### 2.1. Plant Materials and Growth Conditions

*N. benthamiana* plants were grown inside an insect-free growth chamber set at 25 °C and a 16/8 h (light/dark) photoperiod. Plants at the 4–6 leaf stage were used for the experiments.

### 2.2. Vector Construction

For the investigation of the function of SVBV-encoded proteins, a series of plant expression vectors were constructed. Sources of SVBV US isolate (SVBV-US), China isolate (SVBV-SY), Cauliflower mosaic virus (CaMV), SVBV-US infectious clone (pSVBV-US), and expression vectors pCAM-P19, pCAM-2b, pCAM-P1, pCAM-P2, pCAM-P3, pCAM-P4, pCAM-P5, pCAM-P6, and pCAM-P6-GFP were reported previously [3,11,12]. To determine the interaction between SVBV P6 and FveIF3g, the full length of the eukaryotic cell translation initiation factor gene (*FveIF3g*) was PCR amplified from a cDNA that was taken from a total RNA sample isolated from strawberry (*F. Vesca*) leaf tissues. The 3′ terminus of FveIF3g (843 nts) was fused with an mCherry gene through an overlap PCR to generate pF3′ter-mCherry. The full length FveIF3g and F3′ter-mCherry fragments were then inserted individually into the pCAM2300 vector between the *Bam*HI/*Sal*I site to generate p2300-FveIF3g and p2300-F3′ter-mCherry. To explore the mechanism controlling SVBV 35S RNA translation, the full length promoters of SVBV-US and SVBV-SY isolate were also PCR amplified using primer sets pUS-F and pUS-R or pSY-F and pSY-R (Supplementary Table S1). These were used to replace the 35S promoter within the pCAM2300 vector to produce p2300-USpro and p2300-YSpro, respectively. SVBV ORF VII (*p7*) and the 5′ terminal 51 nts of ORF I (5′-*p1*) were PCR amplified using the primer set USP71-F and USP71-R (Supplementary Table S1), and the 3′ terminal of *p7-5′*-*p1* was fused to a *gfp* gene by overlapping PCR to generate pUS-P7-5′-P1:GFP (referred to as US71GFP). Ca71GFP and SY71GFP fragments were also made using the same method. The resulting US71GFP, Ca71GFP, and SY71GFP fragments were released and then inserted into p2300-USpro, p2300-YSpro, or p2300 to produce p2300-USpro-US71GFP (pUSpro-US71GFP), p2300-USpro-Ca71GFP (pUSpro-Ca71GFP), p2300-USpro-SY71GFP (pUSpro-SY71GFP), p2300-YSpro-SY71GFP (pYSpro-SY71GFP), p2300-35S-Ca71GFP (p35Spro-Ca71GFP), and p2300-35S-SY71GFP (p35Spro-SY71GFP), respectively.

To determine the interaction between SVBV P6 and the translation initiation factors of *F. vesca,* the *p6*, *FveIF3g*, *FvL18,* and *FvL24* PCR products were respectively cloned into pGAD-T7, pGBK-T7, p35S::YN, and p35S::YC vectors to generate the constructs pGAD-FveIF3g, pGAD-FvL18, pGAD-FvL24, and pGBK-P6 between the *Eco*RI/*Bam*HI site and YFP^N^-P6, YFP^C^-FveIF3g, YFP^C^-FvL18, and YFP^C^-FveL24 plasmids between the *Sal*I/*Sma*I site. All of the primers used in this study are listed in Table S1, and plasmids were confirmed by sequencing before further use.

### 2.3. Agro-Infiltration and GFP Fluorescence Assay

*Agrobacterium tumefaciens* was transformed with the individual constructs described above and grown overnight in the YEP medium containing the appropriate antibiotics. *A. tumefaciens* cultures were pelleted and then resuspended individually in an infiltration buffer [10 mM MgCl_2_, 10 mM 2-(N-Morpholino) ethane sulfonic acid (MES), and 100 μM acetosyringone]. Individual *A. tumefaciens* cultures were adjusted to *OD*_600_ = 1.2, or as indicated otherwise, and then mixed with the infiltration buffer by 1 to 1 volume for 3 h in dark conditions. The mixed *A. tumefaciens* suspension co-infiltrated into leaves of 7–8 leaf stage *N. benthamiana* plants using 1 mL needleless syringes. The agro-infiltrated leaves were examined for GFP expression under a hand-held 100 W, long-wave UV lamp (UV Products, Upland, CA, USA). The infiltrated leaves were photographed with a Canon EOS 700 D digital camera (Canon, Taiwan, China) with an Y48 yellow filter.

### 2.4. Confocal Microscopy

*N. benthamiana* leaves were infiltrated with *A. tumefaciens* cultures harboring specific combinations of constructs. At 70 h post agro-infiltration (hapi), a leaf disc (1.0 cm^2^) was taken from an infiltrated leaf and examined under an OLYMPUS FluoView™ FV1000 confocal microscope (OLYMPUS, Tokyo, Japan). GFP fusion proteins were excited at 488 nm and the emitted signal was captured at 510 nm. In this study, 4,6-Diamidino-2-phenylindole (DAPI) (Sigma-Aldrich, St. Louis, MO, USA) was used to stain the nuclei in cells. All captured images were processed using the Adobe Photoshop software.

### 2.5. Protein Extraction and Western Blot Assays

The total protein was extracted from *A. tumefaciens* infiltrated leaf samples using the RIPA lysis buffer II (0.5g tissue/mL buffer) as instructed (Sangon Biotech, Shanghai, China). The extracts were centrifuged at 10,000 g for 5 min at 4 °C and the supernatant from each sample was collected. For Western blot assays, the total protein in each supernatant was separated in 10 % SDS-PAGE gels. After transferring protein bands to nitrocellulose membranes, the membranes were probed with a mouse anti-GFP monoclonal or a rabbit anti-P6 polyclonal antibody followed by an HRP-labeled goat anti-mouse IgG or anti-rabbit IgG antibody. Detection signal was visualized using the EasySee Western Blot Kit (TransGen Biotech, Beijing, China).

### 2.6. RNA Extraction and Northern Blot Assays

The total RNA was extracted from agro-infiltrated leaf samples using the Omini Plant RNA Plant Kit (CWBIO, Beijing, China). The synthesis of cDNA was conducted using 1.0 μg total RNA per 20 μL reaction using the PrimeScript™ RT reagent Kit with a gDNA Eraser (TaKaRa, Tokyo, Japan). For Northern blot analyses, the total RNA was first separated in 1.5% denatured agarose gels and then transferred onto Hybond-N+ membranes (GE Healthcare, Chicago, IL, USA). The membranes were hybridized with a DIG-labeled GFP specific DNA probe, and the labeling signal was detected using a labeling and detection starter Kit II (Roche, Basel, Switzerland).

### 2.7. Yeast Two-Hybrid and Bimolecular Fluorescence Complementation (BIFC) Assays 

Interactions between P6 and FveIF3g, P6 and FvL18 or P6 and FvL24 were analyzed through Yeast two-hybrid (Y2H) assay as instructed by the manufacturer (Clontech, Mountain View, CA, USA). The yeast strain Y2H Gold was co-transformed with the pGAD- and pGBK-vectors and grown on an SD-Leu-Trp selective dropout medium for about 48 h in 28 °C. The selected positive colonies were transferred onto the SD/-Ade/-His/-Leu/-Trp plates and incubated at 28 °C for 72 h. For bimolecular fluorescence complementation (BiFC) assays, *N. benthamiana* leaves were co-infiltrated with *A. tumefaciens* cultures harboring YFP^N^- and YFP^C^-vectors using needless syringes. The agro-infiltrated *N. benthamiana* leaves were examined and photographed under the confocal microscope as described above.

## 3. Results

### 3.1. SVBV P6 is a Trans-Activator of RNA Translation

To explore the mechanism controlling SVBV 35S RNA translation, bicistron expression vectors carrying an SVBV-US 35S promoter (pUS-US71GFP) were constructed. The full length ORF VII of SVBV-US served as the first cistron, and its ORF I 5′ terminal 51 nts fused with a *gfp* gene served as the second cistron (Figure 1A). 

To determine which SVBV-encoded protein was a translation trans-activator, we cloned individual SVBV-US ORFs into the pCAM-2300 vector to produce pCAM-USP1, pCAM-USP2, pCAM-USP3, pCAM-USP4, pCAM-USP5, and pCAM-USP6. These vectors were individually co-infiltrated with pUS-US71GFP into *N. benthamiana* leaves. By 3 days post agro-infiltration (dpai), only the leaves co-infiltrated with pCAM-USP6 and pUS-US71GFP showed strong GFP fluorescence (Figure 1B). No strong GFP fluorescence was observed in the leaves co-infiltrated with other constructs. Western blot assays showed a high amount of the GFP protein had accumulated in the leaves co-infiltrated with pCAM-USP6 and pUS-US71GFP. In contrast, only low levels of the GFP protein had accumulated in the leaves co-infiltrated with other constructs (Figure 1C upper panel). Northern blot assays showed that GFP mRNA accumulated at similar levels in all co-infiltrated leaves (Figure 1C).

A previous report showed that the SVBV P6 protein could suppress *gfp* gene silencing triggered by GFP ssRNA [11]. Tombusvirus p19 and CMV 2b are well known silencing suppressors. To exclude the effects of the suppressor functions from the translation, we tested whether the P6 protein could affect the expression of bicistron vectors. When pCAM-p19 or pCAM-2b were co-infiltrated with pUS-US71GFP into *N. benthamiana* leaves, no strong GFP fluorescence was observed by 3 dpai. In contrast, a strong GFP fluorescence was observed in the pCAM-USP6 and US71GFP co-expressed leaves. This indicated that neither the p19 nor the 2b proteins could trans-activate the expression of the GFP protein from the US71GFP bicistron, but the P6 could (Figure S1A). Western blot analyses also revealed that the GFP protein was not detected in the pUS-US71GFP and pCAM-p19 or the pUS-US71GFP and pCAM-2b co-infiltrated leaves (Figure S1B). These results indicate that the function of the RNA silencing suppressor does not promote the expression of the bicistron. Obviously, the P6 protein trans-activation activity is non-related with its VSR activity.

### 3.2. P6 Can Trans-Activate the Translation of the gfp RNA from Different Bicistrons

**Figure 2:**
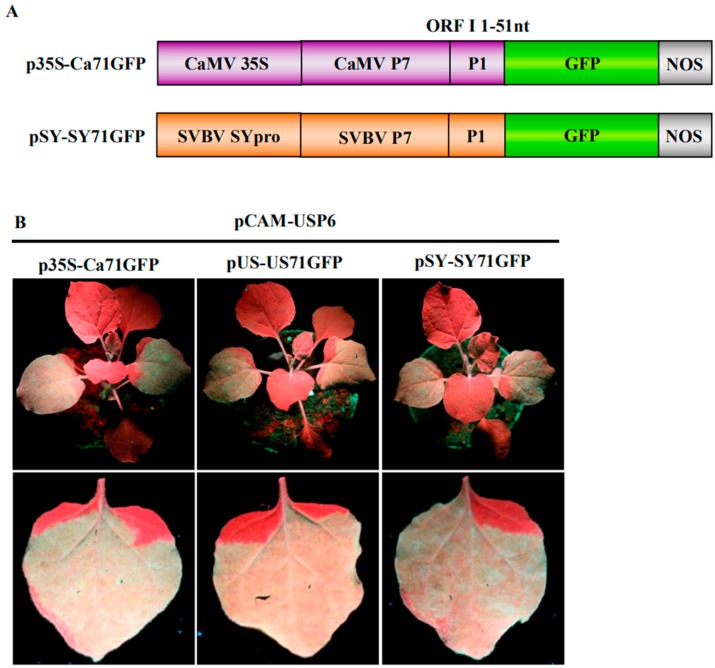

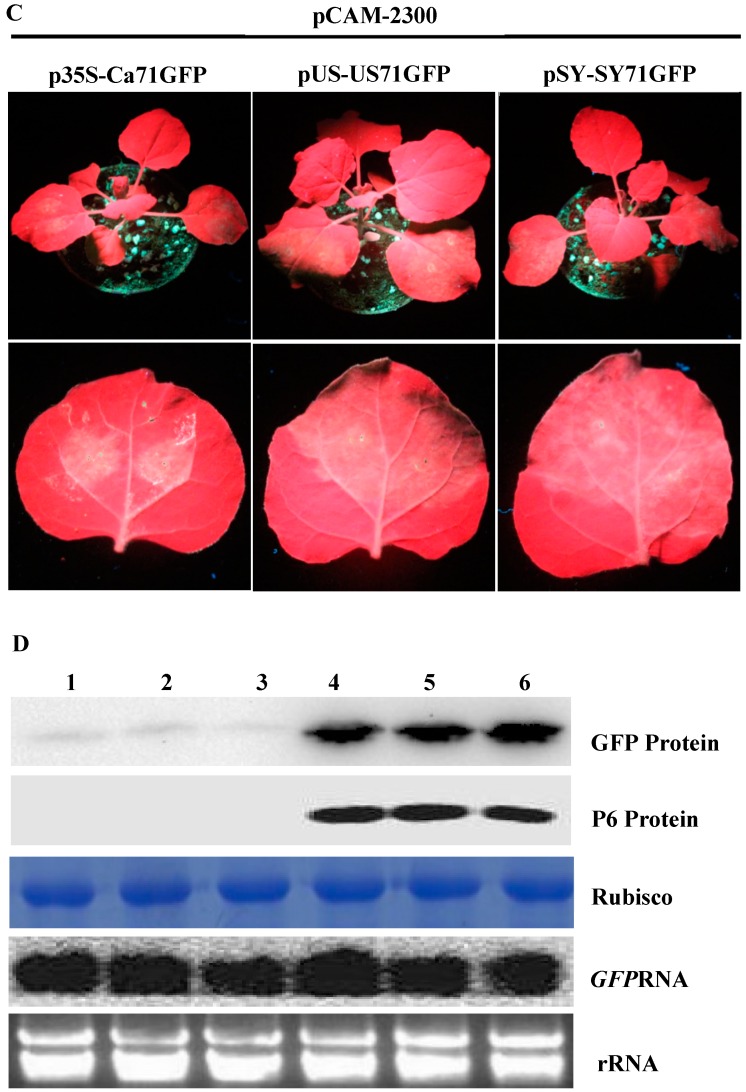
P6 can trans-activate the translation of different bicistrons; (**A**) schematic representations of bicistron constructs. p35S-Ca71GFP contains the CaMV 35S promoter and a fused p7-p1-gfp fragment. pSY-SY71GFP contains the SVBV-SY isolate promoter (pSY) and a fused p7-p1-gfp fragment; (**B**) and (**C**) P6 trans-activated the translations of different bicistrons. *N. benthamiana* plants co-infiltrated with p35S-Ca71GFP, pUS-US71GFP, or pSY-SY71GFP with the empty vector pCAM2300 or with pCAM-USP6 were photographed under the UV illumination at 3 dpai; (**D**) Western blot and Northern blot assays using *N. benthamiana leaves* co-infiltrated with pCAM2300 and p35S-Ca71GFP (lane 1), pCAM2300 and pUS-US71GFP (lane 2), pCAM2300 and pSY-SY71GFP (lane 3), pCAM-USP6 and p35S-Ca71GFP (lane 4), pCAM-USP6 and pUS-US71GFP (lane 5), or pCAM-USP6 and pSY-SY71GFP (lane 6) at 3 dpai. The Western blots were probed with a GFP or SVBV P6 specific antibody. The Northern blot was analyzed as described in Figure 1D).

To further investigate whether the P6 protein can trans-activate the translation of different bicistrons, the CaMV 35S promoter (p35S-Ca71GFP) and the SVBV-SY 35S promoter (pSY-SY71GFP) were constructed. In these vectors, the full length ORF VII of CaMV or SVBV-SY served as the first cistron and their ORF I 5′ terminal 51 nts fused with a *gfp* gene served as the second cistron (Figure 2A). We then used the expression vectors from pUS-US71GFP, p35S-Ca71GFP, or pSY-SY71GFP bicistrons to mimic the expression of proteins from SVBV 35S polycistron RNA through the re-initiation of the translation of downstream cistrons. pCAM-USP6 was co-infiltrated with these three bicistronic constructs into *N. benthamiana* leaves. Leaves co-infiltrated with pCAM-USP6 and an empty vector (pCAM-2300) were used as a negative control. All of the leaves co-infiltrated with pUS-US71GFP and pCAM-USP6, p35S-Ca71GFP and pCAM-USP6, or pSY-SY71GFP and pCAM-USP6 showed strong GFP fluorescence under the UV illumination by 3 dpai. The leaves co-infiltrated with pCAM-2300 and pUS-US71GFP, pCAM-2300 and p35S-Ca71GFP, or pCAM-2300 and pSY-SY71GFP did not show GFP fluorescence (Figure 2B,C). Western blot assays showed that high levels of the P6 and the GFP proteins had accumulated in the leaves co-infiltrated with pCAM-USP6 and pUS-US71GFP, p35S-Ca71GFP, or pSY-SY71GFP. Very low levels of the P6 and the GFP proteins had accumulated in the leaves co-infiltrated with pCAM-2300 and pUS-US71GFP, p35S-Ca71GFP, or pSY-SY71GFP (Figure 2D, Upper panel). Northern blot assays showed that the *gfp* mRNA had accumulated to a similar level in various infiltrated leaves (Figure 2D, Bottom panel). Therefore, we considered that P6 could trans-activate the translation of bicistronic vectors mimicking different—but functionally related—viruses, where the promoter and the first cistron originated from the same virus.

### 3.3. P6 Can Trans-Activate the Translation of gfp mRNA in Bicistrons Expressed Through a Heterozygous Promoter

**Figure 3:**
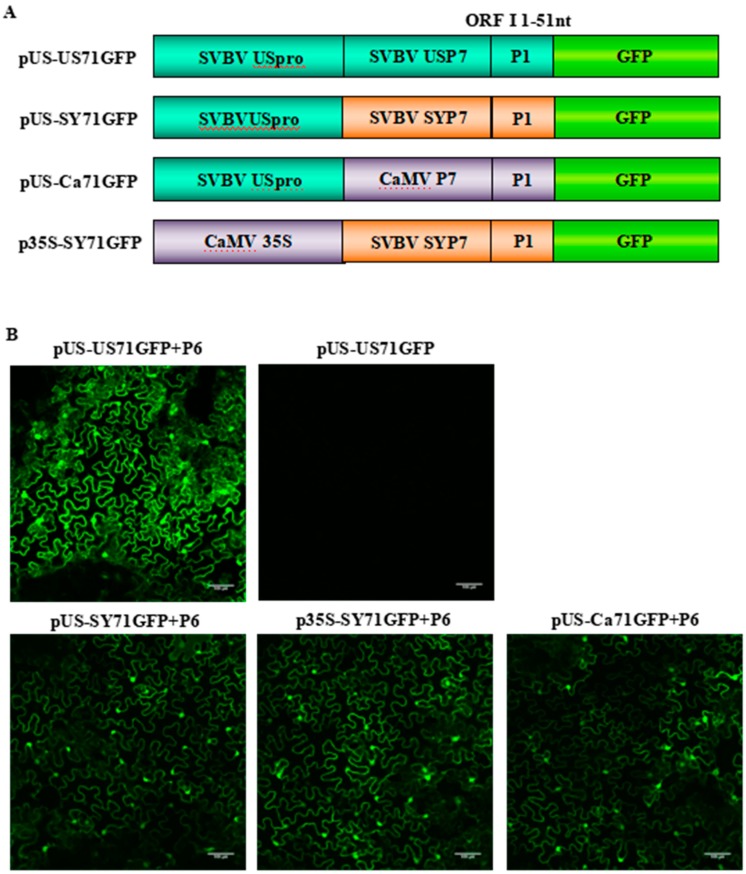
P6 can trans-activate the translation of bicistrons expressed by different 35S promoters; (**A**) schematic representations of four bicistron constructs with different 35S promoters and P7-P1:GFP fragments. The 35S promoters were from SVBV US or CaMV isolate, and the P7-P1:GFP fragments were from SVBV US, SVBV SY or CaMV isolate; (**B**) *N. benthamiana* leaves were co-infiltrated with pCAM-P6 (P6) and pUS-US71GFP, pUS-SY71GFP, p35S-SY71GFP, or pUS-Ca71GFP. Leaves infiltrated with pUS-US71GFP alone were used as negative controls. The infiltrated leaves were examined for GFP expression under a fluorescence microscopy at 70 h post agro-infiltration (hpai). Bars = 100 μm.

To further demonstrate that the P6 protein can trans-activate the translation of *gfp* mRNA, we used bicistrons expressed through heterozygous promoters (i.e., p35S-SY71GFP, pUS-Ca71GFP and pUS-SY71GFP) (Figure 3A). The three constructs were individually co-infiltrated with pCAM-USP6 into *N. benthamiana* leaves. Leaves infiltrated with pUS-US71GFP alone were used as negative controls. The results showed that strong GFP fluorescence was observed in the leaves co-infiltrated with pUS-US71GFP and pCAM-USP6, pUS-SY71GFP and pCAM-USP6, p35S-SY71GFP and pCAM-USP6, or pUS-Ca71GFP and pCAM-USP6 at 70 hpai. In this study, no GFP fluorescence was observed in the leaves infiltrated with pUS-US71GFP alone. Also, among various treatments, the leaves co-infiltrated with pUS-US71GFP and pCAM-USP6 showed the strongest green fluorescence (Figure 3B). These results indicated that SVBV P6 could trans-activate the translation of bicistrons expressed through heterozygous promoters but the trans-activated translation activity was less efficient.

### 3.4. P6 Protein Encoded by SVBV Can Also Trans-Activate the Translation of gfp mRNA

**Figure 4:**
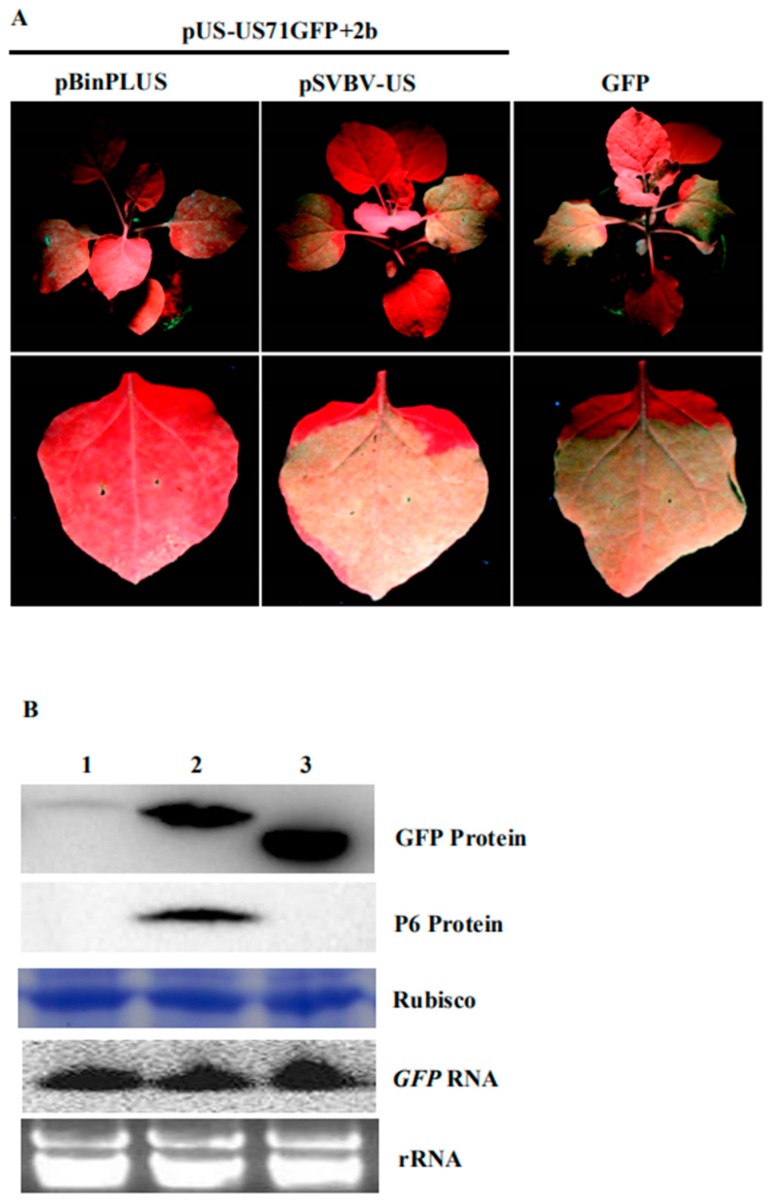
The P6 protein expressed during SVBV infection can trans-activate the translation of a US71GFP bicistron; (**A**) *N. benthamiana* leaves were co-infiltrated with pUS-US71GFP, pBinPLUS, and pCAM-2b (2b), pUS-US71GFP, pSVBV-US, and pCAM-2b (2b), or pCAM-GFP alone (2300-GFP). The infiltrated plants were photographed under the UV illumination at 3 dpai; (**B**) Western blot assay of GFP and P6 protein accumulation, and Northern blot assay of GFP RNA accumulation in the infiltrated leaves. Lane 1, a sample from the pUS-US71GFP, pBinPLUS, and 2b co-infiltrated leaves. Lane 2, a sample from the pUS-US71GFP, pSVBV-US, and 2b co-infiltrated leaves. Lane 3, a sample from the pUS-US71GFP and pCAM2300 co-infiltrated leaves. Procedures of the Western blot and Northern blot assays were the same as described in Figure 1.

*F. vesca* is the original host of the SVBV. Our previous study showed that agro-infiltration of the pSVBV-US infectious clone led to systemic infection, while as *N. benthamiana* is not its host, agro-infiltration with the same clone resulted in local viral protein accumulation only when it was co-infiltrated with a strong suppressor of RNA silencing (CMV 2b) [27]. In contrast, *N. benthamiana* plants agro-infiltrated with the same infectious clone did not show local and systemic infection. Interestingly, when the pSVBV-US infectious clone was co-infiltrated with a vector expressing a CMV 2b protein into *N. benthamiana* leaves, SVBV did accumulate in the infiltrated leaves. Western blot assays using an SVBV P6 specific antibody showed that the P6 protein was present in the infiltrated leaves. To determine if the SVBV-encoded P6 protein could also trans-activate the expression of the *gfp* mRNA in the US71GFP bicistron, we co-infiltrated three constructs (i.e., pUS-US71GFP, pSVBV-US, and pCAM-2b) into *N. benthamiana* leaves. Leaves co-infiltrated with pUS-US71GFP, empty vector pBINPLUS, and pCAM-2b were used as negative controls. Results showed that strong GFP fluorescence was observed in the leaves co-infiltrated with pUS-US71GFP, pSVBV-US, and pCAM-2b by 3 dpai, but not in the negative control leaves (Figure 4A). Western blot assays confirmed that both P6 and GFP proteins had accumulated in the leaves co-infiltrated with pUS-US71GFP, pSVBV-US, and pCAM-2b. Very low amounts of GFP were detected in the control leaves (Figure 4B). Northern blot assays showed that the accumulation of *gfp* RNA in the pUS-US71GFP, pSVBV-US, and pCAM-2b co-infiltrated leaves was similar to that in the negative control leaves (Figure 4B). These results indicated that the translation of US71GFP bicistron could be trans-activated by the SVBV-encoded P6 protein.

### 3.5. P6 Protein Can Interact with FveIF3g of F. vesca Both In Vitro and In Vivo

**Figure 5:**
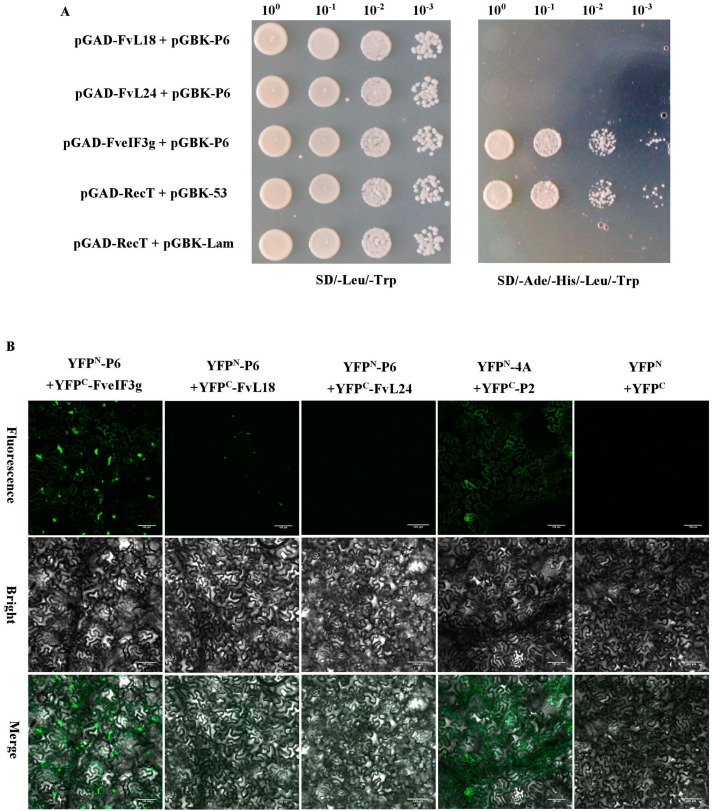

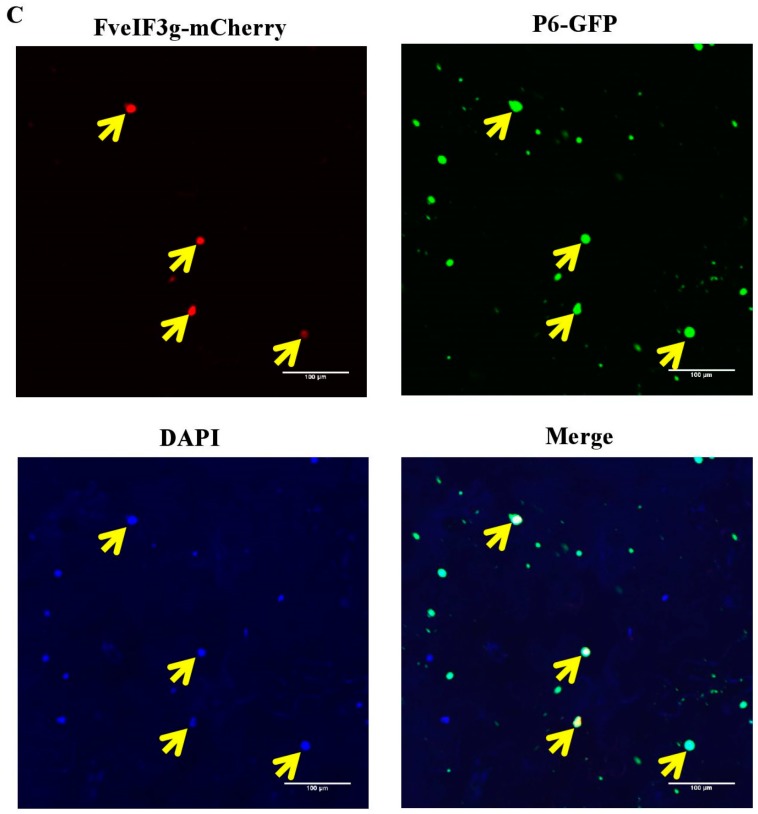
Yeast two-hybrid (Y2H) and BiFC assays for the interaction between the FveIF3g and the P6 protein; (**A**) Y2H gold cells were co-transfected with the indicated plasmids and cultured on the synthetic drop out medium SD-Leu-Trp followed by the SD-Ade-His-Leu-Trp medium. The cells co-transfected with pGAD-RecT and pGBK-53 were used as a positive control and the cells co-transfected with pGAD-RecT and pGBK-Lam were used as a negative control. Dilutions of the transfected cells are indicated at the top of the images; (**B**) leaves of *N. benthamiana* plants were co-infiltrated with various combinations of plasmids: YFP^N^-P6 and YFP^C^-FveIF3g, YFP^N^-P6 and YFP^C^-FvL18, YFP^N^-P6 and YFP^C^-FvL24, YFP^N^-4A and YFP^C^-P2 (positive control), or YFP^N^ and YFP^C^ (negative control). YFP fluorescence was observed by Confocal Microscopy at 70 hpai; (**C**) subcellular localization of FveIF3g-mCherry and P6:CFP in the infiltrated *N. benthamiana* leaf cells was examined and imaged by Confocal Microscopy at 70 hpai. DAPI staining was used to visualize the nuclei in cells. The top left and right images show the FveIF3g-mCherry and P6:CFP fusion fluorescence, respectively. The lower left and right images show the DAPI stained nuclei and the merged image of the three, respectively. Arrows are pointed at the co-localized FveIF3g-mCherry and P6-GFP granules. Bars = 100 µm.

According to previous studies [25], we hypothesized that strawberry polyribosome subunit proteins L18, L24, and initiation factor eIF3g may have roles in the trans-activated translation of SVBV 35S RNA by the P6 protein. *F. vesca FvL18*, *FvL24,* and *FveIF3g* genes were individually cloned into Y2H vectors and then tested for the possible interactions between SVBV P6 and one of the three *F. vesca* proteins through Y2H assays. Results showed that the P6 protein did interact with FveIF3g but not with FvL18 or FvL24 (Figure 5A). Interaction between the P6 and FveIF3g proteins was also confirmed by BiFC in *N. benthamiana* leaves (Figure 5B). When *N. benthamiana* leaves were co-infiltrated with vectors expressing FveIF3g-mcherry or P6-GFP, red fluorescence from FveIF3g-mcherry and green fluorescence from P6-GFP were found to be co-localized in the infiltrated cells at 70 hpai (Figure 5C), suggesting that FveIF3g could interact with the SVBV P6 protein in vivo.

### 3.6. FveIF3g Affects P6 Protein Accumulation and Inhibits the Trans-Activation Activity of P6 Protein

**Figure 6:**
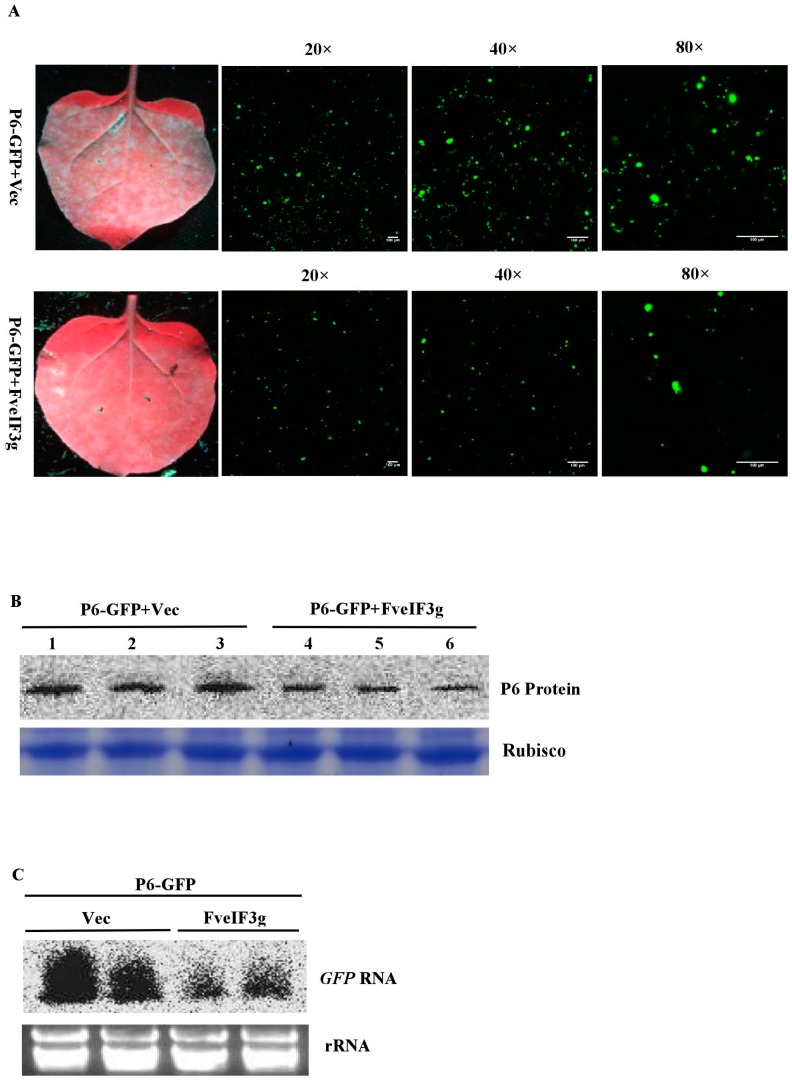

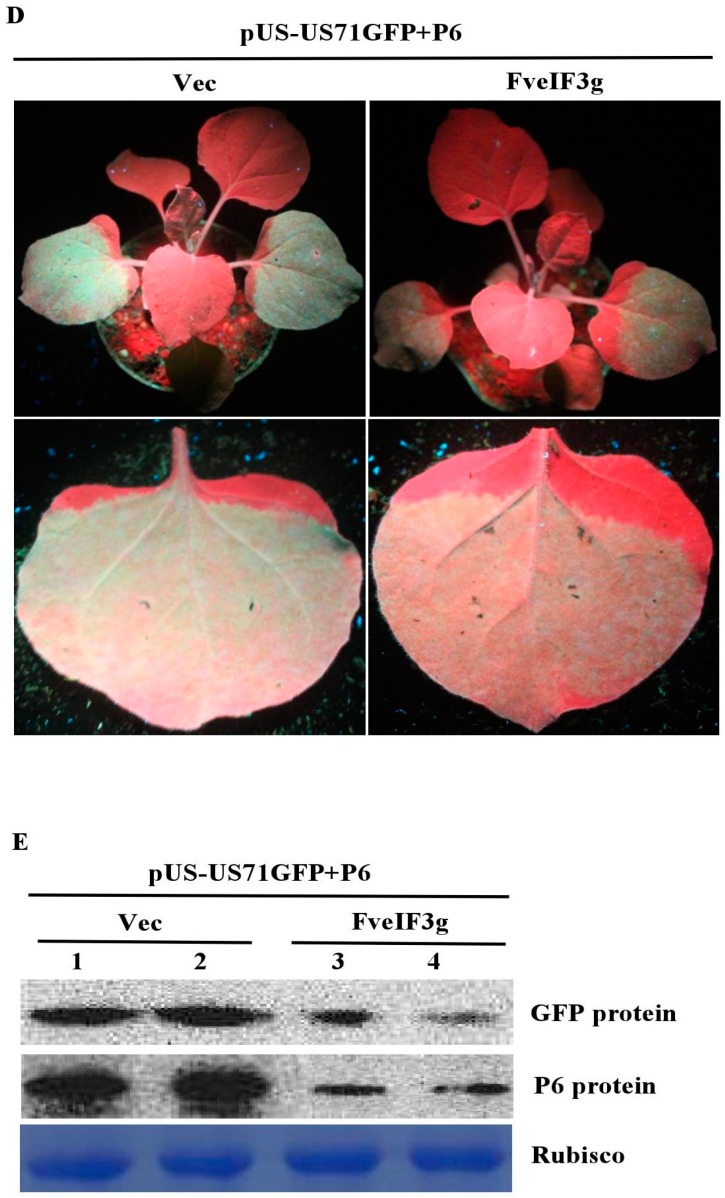
FveIF3g can suppress the accumulation of the P6 protein and its trans-activation activity; (**A**) pCAM-P6:GFP and p2300-FveIF3g (P6-GFP + FveIF3g) or pCAM-P6-GFP and pCAM-2300 (P6-GFP + Vec) were co-infiltrated into *N. benthamiana* leaves. The infiltrated leaves were examined and photographed under the UV light or under a fluorescence microscope at 70 hpai. Bars = 100 μm; (**B**) Northern blot assay of GFP RNA accumulation in the infiltrated leaves; (**C**) Western blot assay of P6 protein accumulation in the infiltrated leaves; (**D**) *N. benthamiana* leaves were co-infiltrated with pUS-US71GFP, pCAM-USP6 (P6), and p2300-FveIF3g (FveIF3g), or pUS-US71GFP, P6, and pCAM-2300 (Vec). The infiltrated plants were photographed under the UV light at 3 dpai. GFP fluorescence in the pUS-US71GFP, P6, and FveIF3g co-infiltrated leaves was weaker than that in the pUS-US71GFP, P6, and Vec co-infiltrated leaves; (**E**) Western blot assays of GFP and P6 protein accumulation in the co-infiltrated leaves at 3 dpai. Coomassie brilliant blue staining was used to estimate the sample loadings.

To test whether FveIF3g could interfere with P6 protein expression, we co-infiltrated *N. benthamiana* leaves with pCAM-P6:GFP and pCAM-FveIF3g or pCAM2300 (control). By 3 dpai, GFP fluorescence was observed in the leaves co-infiltrated with pCAM-P6:GFP and pCAM-FveIF3g. However, the strength of GFP fluorescence was strongly reduced compared with that in the leaves co-infiltrated with pCAM-P6:GFP and pCAM-2300. Confocal microscopy also showed that fewer and smaller P6:GFP granules were present in the cells co-infiltrated with pCAM-P6:GFP and pCAM-veIF3g than in the cells co-infiltrated with pCAM-P6:GFP and pCAM-2300 (Figure 6A, Figure S2). Western blot analyses showed that much less P6 protein had accumulated in the leaves co-infiltrated with pCAM-P6:GFP and pCAM-FveIF3g than had in the control leaves (Figure 6B). Northern blot results also showed that less GFP RNA had accumulated in the leaves co-infiltrated with pCAM-P6:GFP and pCAM-FveIF3g than had in the control leaves (Figure 6C). Taken together, we conclude that FveIF3g can inhibit the accumulation of the P6:GFP fusion protein and the formation of granules in plant cells.

We then examined the effect of FveIF3g on P6 trans-activating activity by co-infiltrating *N. benthamiana* leaves with pUS-US71GFP, pCAM-USP6, and pCAM-FveIF3g or pUS-US71GFP, pCAM-USP6, and pCAM-2300 (control). This was followed by visual observations under the UV illumination at 3 dpai. GFP fluorescence in the leaves co-infiltrated with pUS-US71GFP, pCAM-USP6, and pCAM-FveIF3g was much weaker than it was in the control leaves. Western blot results showed again that the expression level of the P6 protein was much lower in the leaves co-infiltrated with pUS-US71GFP, pCAM-USP6, and pCAM-FveIF3g compared with the control leaves (Figure 6D,E). Taken together, we conclude that the expression of FveIF3g can suppress the P6 protein accumulation, leading to a significant reduction of P6 translation trans-activation activity.

## 4. Discussion

CaMV 35S RNA has a 600 nt leader sequence containing a stem-loop structure in the middle and a short ORF upstream of the stem-loop structure [28]. During virus replication, the ribosome initiates RNA translation along the 35S RNA. After the ribosome slides through the termination codon of the first short ORF, the stem-loop structure will stop the ribosome in order to continue the translation. With the help of CaMV P6, however, the ribosome can scan-reinitiate and shunt to the 3′ end of the stem-loop and then continue to slide through the 35S RNA for downstream ORF translations [29]. The CaMV P6 is also known to have other important roles in virus replication and infection in plants [30].

In this study, we constructed a series of bicistron expression vectors to investigate the mechanism controlling the translation of SVBV 35S RNA polycistron. In these expression vectors, the full length SVBV ORF VII was used to serve as the first cistron and the ORF I 5′ terminal 51 nts fused with a *gfp* gene was used to serve as the second cistron. We reasoned that if the translation of the *gfp* gene in these bicistrons was activated in *N. benthamiana* leaf cells, GFP fluorescence should be observed. Our results showed that the leaves infiltrated with the bicistron expression vector alone did not produce green fluorescence and the GFP protein was not detected in the infiltrated leaves by Western blot assays. When the bicistron expression vector was co-expressed with individual SVBV proteins in *N. benthamiana* leaves, the leaves co-expressing SVBV P6 and the bicistron exhibited strong green fluorescence, and both SVBV P6 and GFP proteins were detected in the infiltrated leaves, indicating that SVBV P6 could trans-activate the translation of the bicistron. In contrast, none of the other SVBV-encoded proteins showed the trans-activation activity in this study.

A previous report indicated that the virus-encoded RNA silencing suppressor could suppress the immune response of a host plant against virus infection, and promote the expression of exogenous genes in plants [31]. Our previous report also showed that the SVBV P6 protein is a multifunctional protein that can act as a viral suppressor of RNA silencing (VSR) to suppress *gfp* gene silencing induced by a single-stranded *gfp* RNA [11]. In this study, we determined that the P6 protein could also trans-activate the expression of the GFP protein when it was co-infiltrated with the pUS-US71GFP bicistron vector into *N. benthamiana* leaves. It is still unclear whether the P6 trans-activation activity is associated with its VSR activity. To investigate this possibility, we compared SVBV P6 with other two VSRs—the TBSV P19 and the CMV 2b protein [32,33]. The results showed that neither the p19 nor the 2b could trans-activate the expression of the GFP protein. This finding indicates that the P6 protein trans-activation activity is independent from its VSR activity.

To further determine the trans-activation activity of SVBV P6, we constructed different bicistron expression vectors driven by different caulimovirus promoters (i.e., pUS-US71GFP, p35S-Ca71GFP, pSY-SY71GFP, p35S-SY71GFP, pUS-Ca71GFP, and pUS-SY71GFP). Agro-infiltration assays showed that the P6 protein was able to trans-activate the translation of all bicistrons, suggesting that the trans-activation ability of the P6 protein was conserved. We speculate that the translation mechanism controlling polycistron translation is the same among caulimoviruses. In this study, we also found that the trans-activation activity of the P6 protein on the bicistrons expressed through the SVBV promoter was stronger than that of the bicistrons expressed through the other promoters. We speculate that this difference is caused by a weaker binding of the SVBV P6 protein to other 35S promoters. Northern blot results confirmed that all the agro-infiltrated leaves accumulated similar amounts of GFP mRNA, indicating that the P6 protein acted at the translation step but not the transcription step.

The SVBV infectious clone, pSVBV-US, could cause local and systemic infection in *F. vesca* but not in *N. benthamiana* plants. In contrast, SVBV infection was observed in the *N. benthamiana* leaves co-infiltrated with pSVBV-US and a vector expressing CMV 2b. In this study, strong GFP fluorescence was observed in the leaves co-infiltrated with pUS-US71GFP, pSVBV-US and pCAM-2b. These results suggest that the translation of bicistron could also be trans-activated by the P6 protein produced during SVBV infection in *N. benthamiana*.

The 80S ribosome is comprised of 40S and 60S ribosomal subunits and can initiate the translation of CaMV 35S RNA during CaMV infection in plants [14]. After finishing the translation of the previous ORF, the 40S ribosomal subunit can maintain the state of translation on the 35S RNA. The CaMV P6 protein reportedly binds the eIF3g and 40S ribosomal subunits, and then recruits the 60S ribosomal subunit to continue the translation of downstream OFRs [34]. Therefore, the interaction between the P6 and eIF3g proteins is needed to start the translation of the later cistrons. In this study, we also investigated the roles of FvL18, FvL24, and FveIF3g during the translation trans-activation by the SVBV P6 protein. Our results showed that only FveIF3g could interact with the P6 and co-localized with the P6 in the nuclei. It is possible that FveIF3g can interfere with ORF VI expression in the nuclei, leading to the reduction of the P6 protein accumulation and the attenuation of the trans-activation of downstream gene translation. The molecular mechanism(s) that cause FveIF3g to inhibit the accumulation of the P6 protein in cells requires future research.

Each plant virus genome encodes at least three proteins. Therefore, all the genomic RNAs of single component RNA viruses are polycistrons. The cistrons located at the 5′ end can be directly translated, but the translation of the cistrons that are far away from the 5′ end need other expression strategies. Subgenomic RNA (Tobamovirus) and polyprotein cleavage (Potyvirus) are the most common strategies for genomic expression in RNA viruses. Caulimoviruses are a kind of dsDNA virus. The polycistron RNAs derived from dsDNA express proteins through trans-activation, which is totally different from subgenomic RNA and polyprotein cleavage. The P6 protein encoded by CaMV ORF VI has been proven to be a trans-activator, and it can efficiently trans-activate the translation of downstream mRNA in a bicistron. Our study showed that the P6 protein encoded by SVBV ORF VI had similar trans-activation function. Interestingly, CaMV ORF VI and SVBV ORF VI share a very low nucleotide sequence similarity—about 40%. However, the SVBV P6 protein not only trans-activated the translation of the SVBV bicistron, but the classical CaMV bicistron as well. This implies that the regions associated with trans-activation in CaMV and SVBV might be very short, possibly controlled by only one or several motifs. The above results indicate that trans-activation might be a common strategy for the genomic expression of caulimoviruses. Our research enriches the understanding of genomic expression strategies of plant DNA viruses.

## Supplementary Materials

The following are available online at http://www.mdpi.com/1999-4915/10/12/717/s1, Table S1: Sequences of primers used for PCR amplifications. Figure S1: Trans-activating activity of CMV 2b and TBSV p19 proteins. Figure S2: The average number of punctuate GFP fluorescent structures in P6-GFP expressed cells compared to P6-GFP and FveIF3g co-expressed cells.

## References

[1] Converse R.H. (1992). Modern approaches to strawberry virus research. Acta Hortic..

[2] Honetšlegrová J., Mráz I., Špak J. (1995). Detection and isolation of *Strawberry vein banding virus* in the Czech Republic. Acta Hortic..

[3] Feng M.F., Zhang H.P., Pan Y., Hu Y.H., Chen J., Zuo D.P., Jiang T. (2016). Complete nucleotide sequence of *Strawberry vein banding virus* China isolate and infectivity of its full-length DNA clone. Virol. J..

[4] Vašková D., Špak J., Klerks M.M., Schoen C.D., Thompson J.P., Jelkmann W. (2004). Real-time NASBA for detection of *Strawberry vein banding virus*. Eur. J. Plant Pathol..

[5] Petrzik K., Benes V., Mraz I., Honetslegrova-Franova J., Ansorge W., Spak J. (1998). *Strawberry vein banding virus*-definitive member of the genus *Caulimovirus*. Virus Genes.

[6] Špak J., Petrzik K. (2006). Variability in sequence of *Strawberry vein banding virus*. Biol. Plant..

[7] Bolton A.T. (1974). Effects of three virus diseases and their combinations on fruit yield of strawberries. Can. J. Plant Sci..

[8] Thompson J.R., Wetzel S., Klerks M.M., Vaskova D., Schoen C.D., Spak J., Jelkmann W. (2003). Multiplex RT-PCR detection of four aphid-borne strawberry viruses in *Fragaria* spp. in combination with a plant mRNA specific internal control. J. Virol. Methods.

[9] Pattanaik S., Dey N., Bhattacharyya S., Maiti I.B. (2004). Isolation of full-length transcript promoter from the *Strawberry vein banding virus* (SVBV) and expression analysis by protoplasts transient assays and in transgenic plants. Plant Sci..

[10] Schoelz J., Shepherd R.J., Daubert S. (1986). Region VI of *cauliflower mosaic virus* encodes a host range determinant. Mol. Cell. Biol..

[11] Feng M.F., Zuo D.P., Jiang X.Z., Li S., Chen J., Jiang L., Zhou X.P., Jiang T. (2018). Identification of *Strawberry vein banding virus* encoded P6 as an RNA silencing suppressor. Virology.

[12] Pan Y., Zhou X.H., Li S., Feng M.F., Shi M.L., Zuo D.P., Jiang X.Z., Ceng J., Hu Y.H., Zhang X.X. (2018). *Strawberry vein banding virus* P6 protein intracellular transport and an important domain identifcation. J. Integr. Agric..

[13] Baughman G.A., Jacobs J.D., Howell S.H. (1988). *Cauliflower mosaic virus* gene VI produces a symptomatic phenotype in transgenic tobacco plants. Proc. Natl. Acad. Sci. USA.

[14] Leh V., Yot P., Keller M. (2000). The *Cauliflower mosaic virus* translational transactivator interacts with the 60S ribosomal subunit protein L18 of *Arabidopsis thaliana*. Virology.

[15] Angel C.A., Lutz L., Yang X.H., Rodriguez A., Adair A., Zhang Y., Leisner S.M., Nelson R.S., Schoelz J.E. (2013). The P6 protein of *Cauliflower mosaic virus* interacts with CHUP1, a plant protein which moves chloroplasts on actin microfilaments. Virology.

[16] Asano K., Clayton J., Shalev A., Hinnebusch A.G. (2000). A multifactor complex of eukaryotic initiation factors, eIF1, eIF2, eIF3, eIF5, and initiator tRNA Met is an important translation initiation intermediate in vivo. Genes Dev..

[17] Hinnebusch A.G. (1997). Translational regulation of yeast GCN4: A window on factors that control initiator-tRNA binding to the ribosome. J. Biol. Chem..

[18] Pestova T.V., Borukhov S.I., Hellen C.U.T. (1998). Eukaryotic ribosomes require initiation factors 1 and 1A to locate initiation codons. Nature.

[19] Pestova T.V., Shatsky I.N., Fletcher S.P., Jackson R.J., Hellen C.U.T. (1998). A prokaryotic-like mode of cytoplasmic eukaryotic ribosome binding to the initiation codon during internal translation initiation of hepatitis C and classical *swine fever virus* RNAs. Genes Dev..

[20] Scholthof H.B., Gowda S., Wu F.C., Shepherd R.J. (1992). The full-length transcript of a *caulimovirus* is a polycistronic mRNA whose genes are transactivated by the product of gene VI. J. Virol..

[21] Zijlstra C., Hohn T. (1992). *Cauliflower mosaic virus* gene VI controls translation from dicistronic expression units in transgenic Arabidopsis plants. Plant Cell.

[22] Hohn T., Park H.S., Peraza O.G., Stavolones L., Pooggin M.M., Kobayashi K., Ryabova L.A. (2001). Shunting and controlled reinitiation: The encounter of *cauliflower mosaic virus* with the translational machinery. Cold Spring Harb. Symp. Quant. Biol..

[23] Tapia M.D., Himmelbach A., Hohn T. (1993). Molecular dissection of the *Cauliflower mosaic virus* translation transactivator. EMBO J..

[24] Ryabova L.A., Hohn T. (2000). Ribosome shunting in the *cauliflower mosaic virus* 35S RNA leader is a special case of reinitiation of translation functioning in plant and animal systems. Genes Dev..

[25] Park H.S., Himmelbach A., Browning K.S., Hohn T., Ryabova L.A. (2001). A plant viral “Reinitiation” factor interacts with the host transcriptional machinery. Cell.

[26] Burks E.A., Bezerra P.P., Le H., Gallie D.R., Browning K.S. (2001). Plant initiation factor 3 subunit composition resembles mammalian initiation factor 3 and has a novel subunit. J. Biol. Chem..

[27] Chen J., Zhang H.P., Feng M.F., Zuo D.P., Hu Y.H., Jiang T. (2016). Transcriptome analysis of woodland strawberry (*Fragaria vesca*) response to the infection by *Strawberry vein banding virus* (SVBV). Virol. J..

[28] Thomas H., Helen R. (2013). Plant pararetroviruses: Replication and expression. Curr. Opin. Virol..

[29] Haas M., Bureau M., Geldreich A., Yot P., Keller M. (2002). *Cauliflower mosaic virus*: Still in the news. Mol. Plant Pathol..

[30] Thiebeauld O., Schepetilnikov M., Park H.S., Geldreich A., Kobayashi K., Keller M., Hohn T., Ryabova L.A. (2009). A new plant protein interacts with eIF3 and 60S to enhance virus activated translation reinitiation. EMBO J..

[31] Zhang X., Yuan Y.R., Pei Y., Lin S.S., Tuschl T., Patel D.J., Chua N.H. (2006). *Cucumber mosaic virus*-encoded 2b suppressor inhibits Arabidopsis argonaute1 cleavage activity to counter plant defense. Genes Dev..

[32] Hsieh Y.C., Omarov R.T., Scholthof H.B. (2009). Diverse and newly recognized effects associated with siRNA binding site modifications on the *Tomato bushy stunt virus* P19 silencing suppressor. J. Virol..

[33] Brigneti G., Voinnet O., Li W.X., Ji L., Ding S., Baulcombe D. (1998). Viral pathogenicity determinants are suppressorsof transgene silencing in Nicotiana benthamiana. EMBO J..

[34] Park H.S., Browning K.S., Hohn T., Ryabova L.A. (2004). Eucaryotic initiation factor 4B controls eIF3-mediated ribosomal entry of viral reinitiation factor. EMBO J..

