# Supplementary material for: Strawberry Vein Banding Virus P6 Protein Is a Translation Trans-Activator and Its Activity Can be Suppressed by FveIF3g
Source: Viruses. 2018 Dec 15;10(12):717. doi: 10.3390/v10120717 (PMC6316418; doi:10.3390/v10120717)
Supplement: Supplementary file 1 [file viruses-10-00717-s001.zip › Supplementary Files/Fig. S1.pdf]

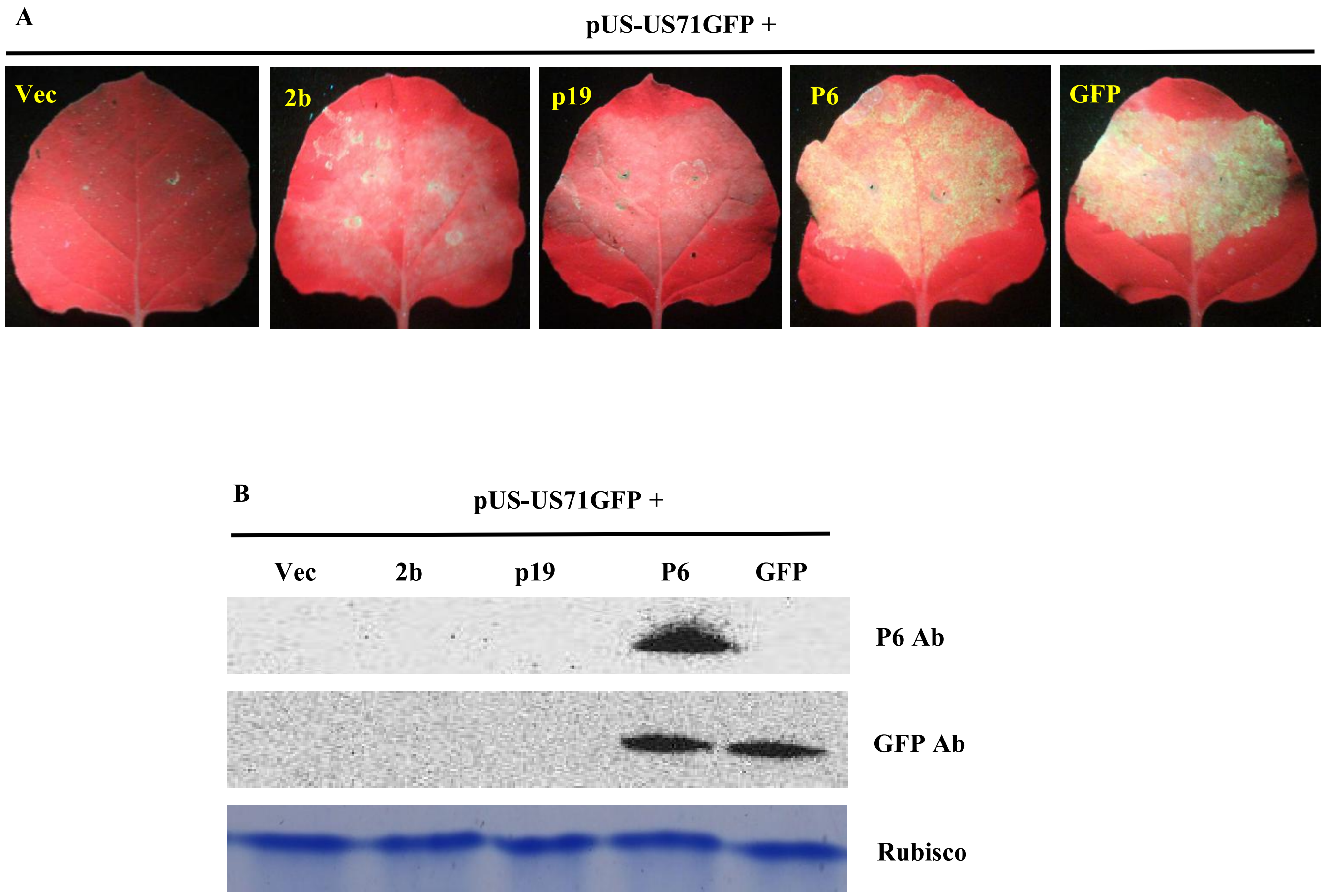

Figure S1. Trans-activating activity of CMV 2b and TBSV p19 proteins. **(A)** Leaves of *N. benthamiana* plants were co-infiltrated with pUS-US71GFP and empty vector pCAM2300 (Vec), pCAM-2b (2b), pCAM-p19 (p19), pCAM-USP6 (P6) or pCAM-GFP (GFP). The infiltrated leaves were photographed under UV light at 3 days post agro-infiltration. (**B**) Western blot assay of P6 and GFP protein accumulations in the infiltrated leaves. The blots were probed with a P6 specific polyclonal or GFP specific monoclonal antibody followed by a HRP-labeled goat anti-rabbit IgG antibody (Ab) or a goat anti-mouse IgG Ab. Coomassie blue staining was used to estimate the protein loadings.
