# Supplementary material for: Strawberry Vein Banding Virus P6 Protein Is a Translation Trans-Activator and Its Activity Can be Suppressed by FveIF3g
Source: Viruses. 2018 Dec 15;10(12):717. doi: 10.3390/v10120717 (PMC6316418; doi:10.3390/v10120717)
Supplement: Supplementary file 1 [file viruses-10-00717-s001.zip › Supplementary Files/Fig. S2.pdf]

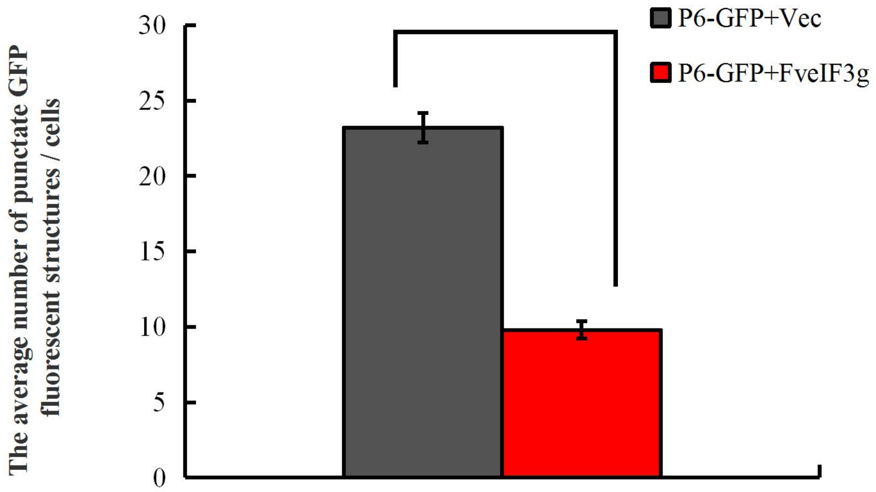

Figure S2. The average number of punctuate GFP fluorescent structures in P6-GFP expressed cells compared to P6-GFP and FveIF3g co-expressed cells. The average number of GFP fluorescent granules counted in individual cells. A total of five cells were counted for each treatment under the fluorescent microscope, and each treatment repeat three times.
