# Supplementary material for: Strawberry Vein Banding Virus P6 Protein Is a Translation Trans-Activator and Its Activity Can be Suppressed by FveIF3g
Source: Viruses. 2018 Dec 15;10(12):717. doi: 10.3390/v10120717 (PMC6316418; doi:10.3390/v10120717)
Supplement: Supplementary file 1 [file viruses-10-00717-s001.zip › Supplementary Files/Table S1.pdf]

Table S1: Sequence of primers used for PCR amplifications.

Primer	Nucleotide sequence (5′-3′)
pUS-F	GAGCTCACTGTGGGTTAATTTC
pUS-R	ACTAGTGACTGGGCGTCTTCAG
pSY-F	GAGCTCACCAAGGACCACTTTG
pSY-R	ACTAGTTTGTAAGCAGGGAGGTTC
USP71-F	ACTAGTATGAGCCATTTCATGAG
USP71-R	CCCGGGGAAGATGTACTCGTCG
SYP71-F	ACTAGTATGAGCCATTTCATGAGC
SYP71-R	CCCGGGGAAGATGTACTCGTCTTG
CaP71-F	ACTAGTATGAATAGGTCTAAGACCAT
CaP71-R	CCCGGGTTGCTCACCATGGATCCCCC
FveIF3g-F	ATGAGACCAGGATCAGCA
FveIF3g-R	TTAATTGGGCCTGAGCCT
FveIF3g (*Bam*HI)-F	GGATCCATGAGACCAGGATCA
FveIF3g-mcherry-F	AGGCTCAGGCCCAATATGGTGAGCAAGGGC
mcherry-FveIF3g-R	GCCCTTGCTCACCATATTGGGCCTGAGCCT
mcherry(*Sal*I)-R	GTCGACACTCGGTACGCGCGGATCTTCCAG
FvL18 (*Eco*RI)-F	GAATTCATGTCAGCGTCTCTT
FvL18 (*Bam*HI)-R	GGATCCTTAGAATTGAAGACCGTT
FvL18 (*Sal*I)-F	GTCGACATGTCAGCGTCTCTT
FvL18 (*Sma*I)-R	CCCGGGTTAGAATTGAAGACCGTT
FvL24 (*Sal*I)-F	GTCGACATGGTTCTCAAGACCGAAC
FvL24 (*Sma*I)-R	CCCGGGTTAGCGCTTACCGCCA
FvL24 (*Eco*RI)-F	GAATTCATGGTTCTCAAGACCGAAC
FvL24 (*Bam*HI)-R	GGATCCTTAGCGCTTACCGCCA

*-Underlined sequences indicate various restriction sites.
